# Human gut microbiome changes during a 10 week Randomised Control Trial for micronutrient supplementation in children with attention deficit hyperactivity disorder

**DOI:** 10.1038/s41598-019-46146-3

**Published:** 2019-07-12

**Authors:** Aaron J. Stevens, Rachel V. Purcell, Kathryn A. Darling, Matthew J. F. Eggleston, Martin A. Kennedy, Julia J. Rucklidge

**Affiliations:** 1 Department of Pathology and Biomedical Science, University of Otago Christchurch, P.O. Box 4345, Christchurch, New Zealand; 2 Department of Surgery, University of Otago Christchurch, P.O. Box 4345, Christchurch, New Zealand; 3 Department of Psychology, University of Canterbury, Christchurch, New Zealand; 4 Mental Health Division, Canterbury District Health Board, Private Bag 4733, Christchurch, New Zealand

**Keywords:** Nutrigenomics, Clinical microbiology

## Abstract

It has been widely hypothesized that both diet and the microbiome play a role in the regulation of attention-deficit/hyperactivity disorder (ADHD) behaviour. However, there has been very limited scientific investigation into the potential biological connection. We performed a 10-week pilot study investigating the effects of a broad spectrum micronutrient administration on faecal microbiome content, using 16S rRNA gene sequencing. The study consisted of 17 children (seven in the placebo and ten in the treatment group) between the ages of seven and 12 years, who were diagnosed with ADHD. We found that micronutrient treatment did not drive large-scale changes in composition or structure of the microbiome. However, observed OTUs significantly increased in the treatment group, and showed no mean change in the placebo group. The differential abundance and relative frequency of Actinobacteria significantly decreased post- micronutrient treatment, and this was largely attributed to species from the genus *Bifidobacterium*. This was compensated by an increase in the relative frequency of species from the genus *Collinsella*. Further research is required to establish the role that *Bifidobacterium* contribute towards neuropsychiatric disorders; however, these findings suggest that micronutrient administration could be used as a safe, therapeutic method to modulate *Bifidobacterium* abundance, which could have potential implications for modulating and regulating ADHD behaviour. Our pilot study provides an initial observation into this area of research, and highlights an interesting avenue for further investigation in a larger cohort. Furthermore, these novel results provide a basis for future research on the biological connection between ADHD, diet and the microbiome.

## Introduction

Understanding the molecular interactions between nutrition, and mental health and disease is an important challenge for medical biology^1^. A growing body of evidence indicates that nutritional components impact on both physical and mental health, suggesting that specific dietary manipulations may be a useful form of treatment for some diseases^2–4^. Dietary supplementation with a broad spectrum of micronutrients (vitamins, minerals, amino acids) is one such treatment option that is gaining support in the scientific literature, with demonstrated benefits in modulating attention-deficit/hyperactivity disorder (ADHD), mood, anxiety, stress, aggression and symptoms associated with autism^5–8^. However, there is little scientific research into the biochemical effects of dietary supplements or investigation into the potential biological pathways through which they may act^9^.

It has been hypothesized that alterations in the human gut microbiota by micronutrients^10–15^, may have a biochemical effect via the vagus and spinal nerves^16^, which enable bidirectional communication between the gut and the brain, referred to as the gut-brain axis^17,18^. Through this pathway, metabolites can act as modulators in neural, immunological and hormonal signalling, which have important implications for health and disease^19–21^. Although the exact mechanisms are not fully understood, diet is thought to be one of the most influential factors on the microbiome^22–24^.

The microbiome has been observed to play an important role in anxiety-like behaviour in animal studies^22,25^, and this has been observed in human patients with irritable bowel syndrome^26,27^. There is also growing evidence supporting a role of the human gut microbiome in the development of neuropsychiatric disorders, such as depression^4,18^, ADHD and autism^28–30^. ADHD is a developmental disorder that affects approximately 7.2% of children^31^, where pharmacological treatments can reduce symptoms, but are often unsatisfactory due to side effects, failure to prevent or alter long-term course and discontinuance due to patient and family preferences^32^. Growing evidence is suggesting that nutrition plays an important role in ADHD behaviour^33,34^, with long-term studies showing that early malnutrition is an important risk factor^35^.

We performed a pilot study investigating the potential effects of micronutrients on human faecal microbiome content. This study was carried out in a sample of children between the ages of seven and 12 years, diagnosed with attention-deficit/hyperactivity disorder (ADHD). These individuals were a subset of children who had participated in a 10 week double-blind RCT comparing a broad-spectrum micronutrient treatment with a placebo to investigate therapeutic effects on ADHD symptoms^36^. In this study, micronutrient administration was associated with improved overall function, reduced impairment and improved attention, emotional regulation and aggression relative to placebo^36^.

## Methods

### Randomized control trial layout

**Figure 1:**
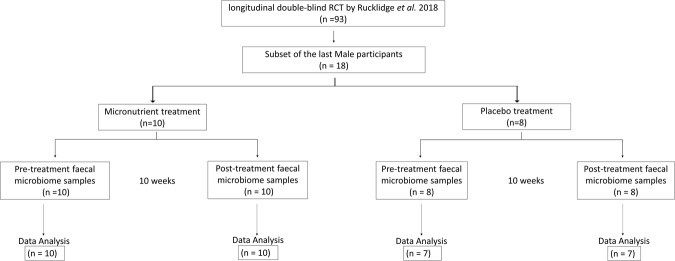
Study design and RCT layout. The 10-week pilot study was performed with a sub-sample of participants from a larger study^36^. Study design followed a standard double-blind, randomized control trial (RCT), and there was no *a priori* selection of candidates for participation in the microbiome analysis.

This 10-week pilot study followed a standard double-blind, randomized control trial (RCT) design, where participants were randomized to either placebo or control. The cohort consisted of 18 consenting children diagnosed with ADHD, who were a sub-cohort of participants from a larger study that investigated the effects of dietary micronutrient supplementation on ADHD outcomes^36^. Faecal microbiome analysis was not intended at the initialisation of this large study^36^, and this sub-cohort represents the later male recruits. There was no *a priori* selection of candidates for participation in the microbiome analysis (Fig. 1) and the randomization was not influenced.

The placebo group contained eight participants and the micronutrient group contained ten participants. One participant in the placebo group had taken oral antibiotics during the RCT and was subsequently excluded at the data analysis stage, giving a final total of 34 samples (Fig. 1). For each participant, a pre-RCT, and a post-RCT faecal sample was collected, and microbiome sequencing analysis was performed on DNA extracted from each sample, giving a total of 36 samples (Supplementary File 1).

All participants were under supervised administration of capsules, where the micronutrient group received a formulation containing a blend of vitamins, minerals, amino acids and antioxidants (see Supplementary Table 1 for ingredients of both treatment and placebo capsules). Participants began by taking one capsule, three times each day, increasing the dose by three capsules every two days up to a target dose of 12 capsules per day: four taken at three different intervals. Further information regarding the dosing, randomization and blinding procedures is available in Rucklidge *et al*.^36^.

### Statistical design

Due to the RCT layout of this study, the dataset consisted of paired samples for each individual that had been collected pre and post-RCT. Where possible, the data were analyzed using a paired-sample approach to appropriately incorporate the baseline data for each individual. This ensured that the study specifically investigated changes that occurred during the trial period. Significant changes were only considered true, if they were specific to the micronutrient-treatment, or if the magnitude of change was in the opposite direction to the placebo. In addition to the below methods, repeated measures correlation was performed using the R package, rmcorr^37^, and power analysis was performed using the R package, PWR^38^. Correlation analysis of the abundance data was performed within R, using the “Pearson” correlation coefficient.

### Efficacy and safety assessments

All participants were monitored by a clinical psychologist or psychology graduate student, under the supervision of a psychologist. This was performed at the screening visit, baseline, and weeks 2, 4, 6, 8 and 10 (or end of study), via face-to-face meetings or via phone contact. At each visit, the Children’s Global Assessment Scale (CGAS)^39^ and ADHD Rating Scale IV (ADHD-RS-IV) – clinician^40,41^ were completed. The CGAS was used by the clinician to assess the overall level of the children’s functioning based on all the information gathered since the last visit. Scoring is based on a single numerical scale from 1 to 100, where the higher score is indicative of better functioning. At pre and post-RCT time-points, the clinician also completed the ADHD-RS-IV, which contains 18 items directly linked to DSM-IV diagnostic criteria for ADHD and provides a total score and two subscale scores for inattention and hyperactivity/impulsivity, assessing ADHD symptoms based on frequency (0 ‘never or rarely’ to 3 ‘very often’). The clinician took into account observations from visits and formal cognitive testing, information from others, as well as parent report in determining ratings. However, frequency of behaviours was the main focus of the rating, considering how often the behaviours were present.

At baseline and study completion, hematological variables, biochemical variables, thyroid function, prolactin, fasting glucose, homocysteine, iron, zinc, vitamin D, vitamin B12, copper, blood pressure, height and weight were also recorded. To assess the child’s dietary patterns, including consumption of fruit, vegetables, breakfast, and fast foods, a brief diet intake questionnaire was performed at baseline and end of RCT with a higher score indicative of a healthier eating pattern (modified from Baker, *et al.*^42^). Demographic variables, including participant’s ethnicity and parents’ occupation were also collected at baseline.

### Informed consent and ethics

Written informed consent was obtained from all of the participants’ parents or legal guardians and assent was obtained from the participants. The trial was prospectively registered with the Australia and New Zealand Clinical Trial Registry ACTRN12613000896774, on the 12/08/2013. The exploratory nature of the study, as well as other treatment options for ADHD, was explained to participants and their parents prior to enrolling. This study was approved by the University of Canterbury (New Zealand) and national institutional review boards. Ethical approval for this study was approved by the Southern Health and Disability Ethics Committee (New Zealand), Ethics ref: 13/STH/45/AM05, and all research was performed in accordance with the relevant guidelines.

### Sample collection and storage

Fresh stool samples were collected at baseline and post RCT (10 weeks) using the OmnigeneGut faecal collection system (DNA Genotek, Ottawa, Canada), according to the manufacturer’s specifications. Using this system, homogenised samples are stable for 60 days at ambient temperature, however, all samples were stored at −4 °C for a maximum of 14 days, before aliquoting and storage at −80 °C.

### DNA Isolation and sequencing

Total DNA was extracted from stool samples using the NucleoSpin DNA Stool isolation kit by Macherey-Nagel (Germany, Duren), according to the manufacturer’s recommendations for human stool samples. Extracted DNA was stored at −20 °C until PCR amplification and Illumina MiSeq DNA sequencing. PCR amplification and single-end DNA sequencing of the V3/V4 regions of the 16S rRNA gene was outsourced to New Zealand Genomics Limited (Dunedin, New Zealand) and performed according to their protocols.

### Bioinformatics analysis

Demultiplexed and pre-processed sequence reads were supplied by New Zealand Genomics Limited and these were imported into the Quantitative Insights Into Microbial Ecology (QIIME2, version 2017.12), python-based pipeline using CASAVA 1.8. Barcode and primer removal, quality control, amplicon sequence data correction, phiX filtering, and dereplication was verified using the DADA2 software package^43^. The total sequence length was truncated to 220 bp, and 13 bp of low quality data were trimmed from the start of the sequence. This processing was separately performed on the V3 and V4 sequence runs. Feature tables and representative sequence files were then merged for downstream analysis, within QIIME2.

### Taxonomic classification

Taxonomic classification was assigned using the GreenGenes database, specific for the V3/V4 16S region (version 13.8). All taxonomic classifications were implemented within QIIME2 and assigned using the naïve Bayesian algorithm, developed for sklearn classifier. For phylogenetic diversity analysis, sequences were aligned using the MAFFT^44^ programme plugin, and then filtered to remove highly variable positions. Reads that were unassigned at the Kingdom level were removed from the OTU table, as were OTUs that were observed less than 100 times (Supplementary File 2). The final taxonomic classification was made against the filtered feature table, which had 4474 distinct features (Supplementary File 3).

### Phylogenetic analysis

Alpha and Beta diversity were calculated using the q2-diversity plugin and included Faith’s Phylogenetic Diversity, and weighted and unweighted Unifrac distances. The feature table was rarefied to a sampling depth of 273,465, which retained 50.18% (﻿﻿9,297,810) of sequences and all samples (34). This sampling depth was selected as it was approaching the maximum depth which retained all samples for our analysis. Principal coordinate analysis (PCoA) was used to investigate the similarities between bacterial communities, based on treatment using Unweighted and Weighted Fast Unifrac methods. Pairwise distance and pairwise differences in alpha diversity values (observed operational taxonomic units) were calculated within QIIME2, using the q2-longitudinal plugin.

### Computing species abundance using the q2-ANCOM plugin

Species abundance was assessed using the QIIME2 ANCOM plugin^45^, consecutively at each taxonomic level to detect significant changes in abundance between each of the four groups (micronutrient pre-RCT, micronutrient post-RCT, placebo pre-RCT and placebo post-RCT). Relative frequencies at each phylogenetic level were calculated by ANCOM, from the DADA2 feature table for assigned sequence variant.

### Analysing paired differences in abundance using the q2-longitudinal plugin

Longitudinal analysis of feature data from paired samples (pre and post-RCT) was performed following the methods outlined by Bokulich *et al*.^46^. Briefly, relative abundance was manually calculated from the DADA2 generated (non-rarefied) feature table, filtered to contain Actinobacteria, and consecutively collapsed at each phylogenetic level (Supplementary File 4 and 5). The pairwise difference between baseline and post-RCT samples for each phylogenetic level was then compared for the micronutrient and placebo groups. This method measures within group pairwise difference using Wilcoxon signed-rank tests, and pairwise group comparison tests using Kruskal Wallis.

### Functional predictions

Functional predictions of metagenomics profiles were computed using two common approaches. The first approach involved analysis with the R package Tax4Fun^47^, and the second approach involved analysis with the package, Phylogenetic Investigation of Communities by Reconstruction of Unobserved States (PICRUSt)^48^, via the Galaxy platform^49^. For analysis with Tax4Fun, a QIIME2 closed reference feature table was generated using the QIIME2 vsearch plugin at 97% similarity to the SILVA^50,51^ database release version 132. For analysis using PICRUSt, the feature table was generated at 97% similarity to the Green Genes database version 13.5.

Change in the abundance of metagenomic pathway profiles generated by each approach was then calculated by subtracting the baseline measurement from the post-RCT measurement, for each individual. Differentially abundant pathways that correlated with treatment were identified using a linear regression model within the Limma package^52^ that accounted for treatment, age, and ethnicity.

## Results

### Participants

Participants were males who ranged from seven to 12 years (Table 1) and consisted of 13 New Zealand Europeans and four New Zealand Māori participants. The groups were well matched and there were no group differences at baseline in severity of ADHD behaviours, socio-economic status, body mass index, dietary patterns, or presence of other comorbid disorders. There were no significant differences between the subsample and the larger cohort for the measured variables presented in Table 1(p > 0.05). Adherence to treatment regimen was measured by parent reports and returned treatment capsule counts. Parent reports indicated adherence for the micronutrient group was 95.3% (+/−6.3), and 93.4% +/−7.6) for the placebo group. Capsule counts indicated that adherence in the micronutrient group was 89.94% +/−17.2) and 91.0% +/−7.3) for the placebo group. The large standard deviation in the micronutrient group resulted from one participant and adherence calculated in the absence of this participant was 95.7% +/−3.4). Because this is a pilot study, and the concentration required for microbiome modification is unknown, this individual was not excluded from the analyses. The response rate in this study was consistent with that reported in the large cohort, with 50% responders (5/10) in the treatment group versus 29% (2/7) in the placebo group. However, there was no significant difference between ADHD-IV-RS and CGAS metrics in the two groups. Further information regarding participants and eligibility is available in Rucklidge *et al*.^36^. The diet intake questionnaire indicated that there was no significant difference in the recorded dietary habits between pre and post RCT for either group, with no significant group difference at post-RCT, after controlling for pre-RCT (*F*_1,15_ = 0.060, *p* = 0.81).

**Table 1:** Clinical description of population.

	Control	Treatment
	n = 7	n = 10
**Population variables (standard deviation)**		
Body mass index	16.6 (3)	19.39 (2.9)
Age	9.3 (1.3)	10.29 (1.9)
ADHD symptoms*	44.6 (4)	41.60 (11.3)

*Based on clinician ratings using the ADHD-IV-RS.

### Sequence processing

Files were processed using the DADA2 package, after which there were 18,976,535 sequences with 4,474 sequence variants from all samples. The mean sequence frequency per sample was 558,133 and all samples had a read number greater than 285,765.

### Classification of OTU

OTUs were classified using QIIME2 and the.biom file was imported into the R statistical package, Phyloseq for further analysis (summarised in Table 2). For all sequence variants, bacteria accounted for 87% of assigned variants. The main phyla in all individuals was Bacteroidetes with a relative abundance of 46.8% +/− 3.9). The three next most abundant phyla were Firmicutes (45.3%, +/− 2.2), Proteobacteria (0.04%, +/− 0.05) and Actinobacteria (0.03%, +/−0.02). For both the micronutrient and placebo groups the dominant phyla shifted from Firmicutes to Bacteroidetes between the pre- and post-RCT time points, where Firmicute abundance remained consistent. Although Actinobacteria only accounted for a small portion of the top phyla, there was a substantial decrease in abundance observed in the post-RCT, micronutrient group (Table 2). For the relative frequency of all bacteria detected at the phylum level see Supplementary Fig. 1 and 5, and for the relative frequency of all bacteria detected at the genus level see Supplementary Fig. 2. For further visualisation of the relative frequencies for the full dataset, Supplementary File 4 can be uploaded into the interactive visualisation tool available at https://view.qiime2.org/.

**Table 2:** Relative frequency of each major phylum.

Group	Bacteroidetes	SD +/−)	Firmicutes	SD +/−)	Proteobacteria	SD +/−)	Actinobacteria	SD +/−)
Baseline micronutrient	11.36	0.34	13.72	0.54	1.02	0.06	0.98	0.08
Post micronutrient	16.65	0.41	12.74	0.22	1.86	0.10	0.28	0.02
Baseline placebo	7.16	0.29	9.13	0.22	0.66	0.05	0.67	0.04
Post placebo	11.41	0.41	9.90	0.42	1.24	0.10	0.60	0.02

*Summary of (non-rarefied) frequency table generated by QIIME2

### Alpha and beta diversity analysis

**Figure 2:**
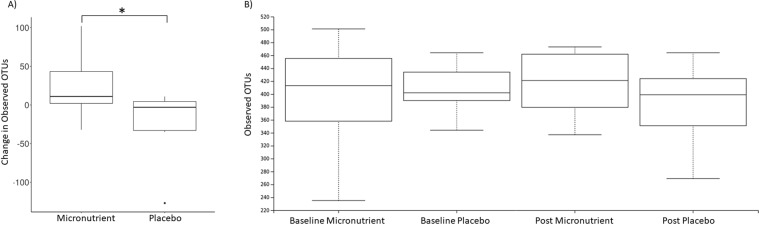
Pairwise difference plots for Alpha and Beta diversity metrics. Treatment group is represented on the x-axis and pairwise change between pre and post measurements are demonstrated on the y-axis. The asterisk denotes significance (*p* = 0.05). (**A**) Pairwise difference comparison in observed OTUs (Alpha diversity). (**B**) Pairwise difference comparison of community richness using Shannon indices (Beta diversity).

**Figure 3:**
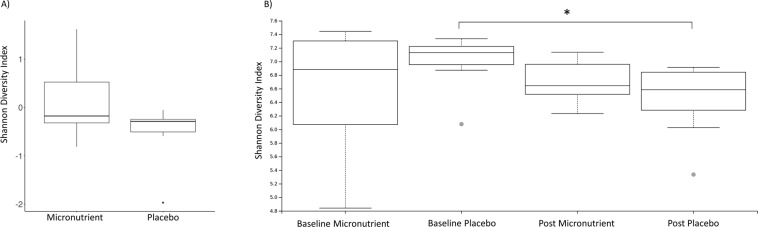
Pairwise difference plots for Alpha and Beta diversity metrics. Treatment group is represented on the x-axis and pairwise change between pre and post measurements are demonstrated on the y-axis. The asterisk denotes significance (adj*p* = 0.05). (**A**) Pairwise difference comparison in observed OTUs (Alpha diversity). (**B**) Pairwise difference comparison of community richness using Shannon indices (Beta diversity).

The within sample phylotype richness (alpha diversity) and dissimilarity (beta diversity) were calculated from the rarefied frequency table. The pairwise differences were then calculated by subtracting pre-RCT measures from the post-RCT for each individual. This enabled the comparison of changes at each stage (pre and post) and between each group (micronutrient and placebo). This was performed within the QIMME2 longitudinal plugin which is specifically optimised for analysis of paired samples. There was a significant difference in the change of observed OTUs between the treatment and placebo groups (Kruskal Wallis test *p* = 0.05, *H* = 3.8) (Fig. 2A). However, within each group (between baseline and post-RCT) there was no significant change (*p* > 0.2) (Fig. 2B). There was no significant quantitative change between the placebo and micronutrient treatments, as assessed using Shannon diversity index (Kruskal Wallis *p* > 0.1, *H* = 1.4), which is a quantitative measure of species diversity (Fig. 3A). However, the placebo group demonstrated a significant decrease in community richness between pre and post-RCT (Wilcoxon signed-rank test, adj*p* = *0*.*04*) (Fig. 3B). There was no significant change in beta diversity between pre- and post-RCT, assessed using weighted and unweighted unifrac distance matrices (data not shown).

### Differential abundance

**Figure 4:**
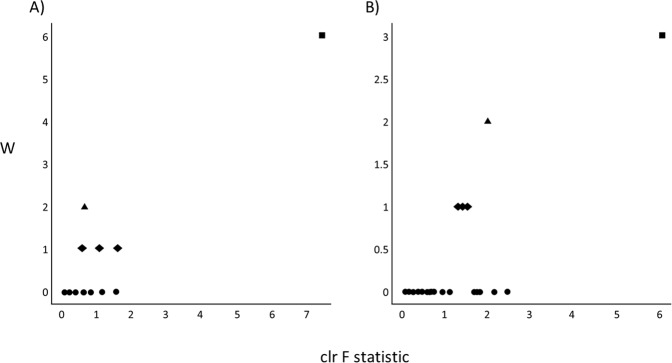
Differentially abundant microbial taxa identified by ANCOM. Volcano plot of differential abundance at the group level (pre-RCT placebo, pre-RCT micronutrient, post-RCT placebo and post-RCT micronutrient). F-statistics are represented on the x-axis and W-statistics on the y-axis. The F-statistics are a measure of the effect size difference for a particular species between the study groups, and the W-statistic is the strength of the ANCOM test for the tested number of species. (**A**) Differential abundance at the phyla level, the square represents Actinobacteria, the triangle represents Bacteroidetes, the diamonds represent Firmicutes, Proteobacteria, and Fusobacteria, circles represent Lentisphaerae, cyanobacteria, Verrucomicrobia, Tenericutes, Euryarchaeota and TM7. (**B**) Differential abundance at the order level, the square represents Bifidobacteriales, the triangle represents Fusobacteriales, diamonds represent Pasteurellales, Burkholderiales and Bacteroidales, and circles represent Cerasicoccales, YS2, Actinomycetales, Clostridiales, Victivallales, Verrucomicrobiales, Campylobacterales, Erysipelotrichales, Gemellales, RF32, Desulfovibrionales, Enterobacteriales, Lactobacillales, ML615J-28, Methanobacteriales, Anaeroplasmatales, Turicibacterales, RF39, Coriobacteriales, and Streptophyta.

We next investigated how the micronutrient treatment influenced the relative abundance of microbial features at each time point. We first used Analysis of Composition of Microbiomes (ANCOM)^45^ implemented through the QIIME2 ANCOM plugin in order to identify features that significantly differed in abundance from one or more of the four groups (micronutrient pre-RCT, micronutrient post-RCT, placebo pre-RCT and placebo post-RCT). The ANCOM algorithm determines significance by plotting calculated F-statistics on the x-axis and W-statistics on the y-axis. The F-statistics is a measure of the effect size difference for a particular species between the study groups, and the W-statistic is the strength of the ANCOM test for the tested number of species. In total 11 phyla were identified: Euryarchaeota, Actinobacteria, Bacteroidetes, Cyanobacteria, Firmicutes, Fusobacteria, Lentisphaerae, Proteobacteria, TM7, Tenericutes, and Verrucomicrobia; however, Actinobacteria were the only phylum to demonstrate a significant change in response to treatment (*W* = 6, *clr f statistic* = 7.5) (Fig. 4A). This observation was consistent for the taxonomic classification levels of class (data not shown) and order, and at the order level the effect was attributed to a decrease in the abundance of Bifidobacteriales (*W* = 3, *clr f statistic* = 6.04) (Fig. 4B). Marginally significant changes in differential abundance were also observed for the orders Fusobacteriales (*W* = 2, *clr f statistic* = 2.1), Bacteroidales (*W* = 1, *clr f statistic* = 1.5), Pasteurellales (*W* = 1, *clr f statistic* = 1.3) and Burkholderiales (*W* = 1, *clr f statistic* = 1.4) (Fig. 4B). However, for these weaker observations, analysis of the raw data indicated that the effect was either not specific to the treatment, or that the statistical analysis was likely to be biased by low abundances of assigned sequence variants. Therefore, these observations were not further pursued in subsequent analyses.

### Longitudinal analysis of relative frequency

Because ANCOM does not currently support pairwise analysis of samples within QIIME2, we further investigated the magnitude of changes by plotting the relative bacterial frequency for each individual using the q2-longitudinal plugin for pairwise differences. For this analysis, the relative frequency (as opposed to differential abundance) of assigned sequence variants were calculated from the DADA2 feature table (Supplementary File 5), and significance was assessed by comparing the pairwise difference between baseline and post-RCT samples for the micronutrient and placebo groups.

**Figure 5:**
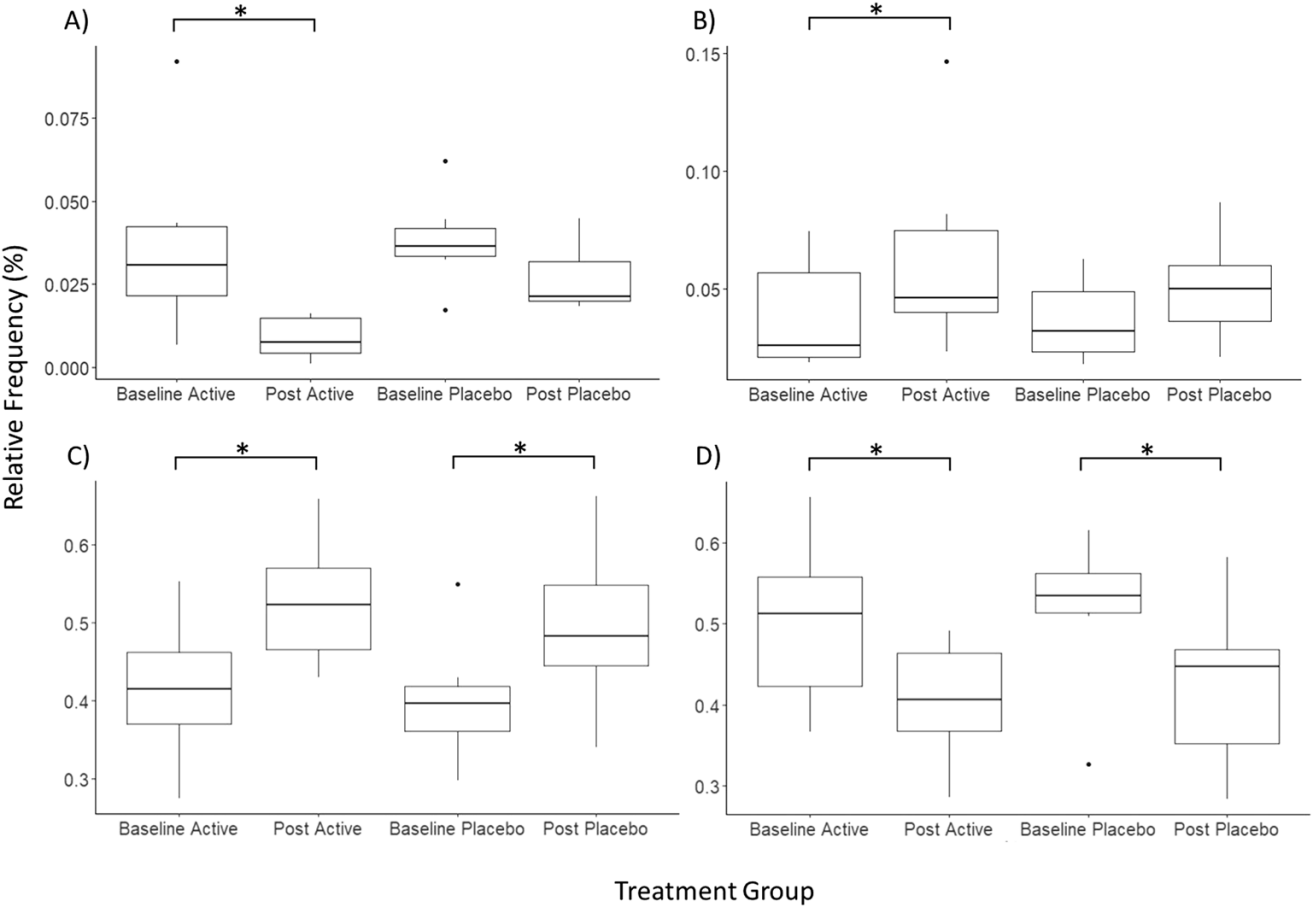
Phyla level comparison in pairwise relative frequency at each treatment stage. The y-axis corresponds to relative frequency (as percentage), and treatment group is presented on the x-axis. The asterisk denotes a significant change (adj*p* ≤ 0.05) between pre and post-RCT groups. (**A**) Actinobacteria (**B**). Proteobacteria (**C**). Bacteroidetes (**D**). Firmicutes.

In the post-micronutrient treatment group, Actinobacteria frequency significantly decreased by approximately 2% (adj*p* = 0.01). Although a decrease was also observed in the placebo group for several individuals, the pairwise difference did not reach significance (adj*p* = 0.06) (Fig. 5A). Proteobacteria demonstrated a minor, but significant increase in the micronutrient group, but not the placebo group (micronutrient adj*p* = 0.05, placebo adj*p* = 0.2);. However, the raw data had high inter-individual variability and was substantially influenced by a single outlier in the micronutrient group, which is likely to be why ANCOM did not attribute this effect to the micronutrient treatment (Fig. 5B). This effect was therefore not investigated at lower taxonomic levels. Bacteroidetes abundance significantly increased in both groups (micronutrient adj*p* = 0.018, placebo adj*p* = 0.018) (Fig. 5C), and Firmicute abundance significantly decreased in both groups (micronutrient adj*p* = 0.018, placebo adj*p* = 0.018) (Fig. 5D).

**Figure 6:**
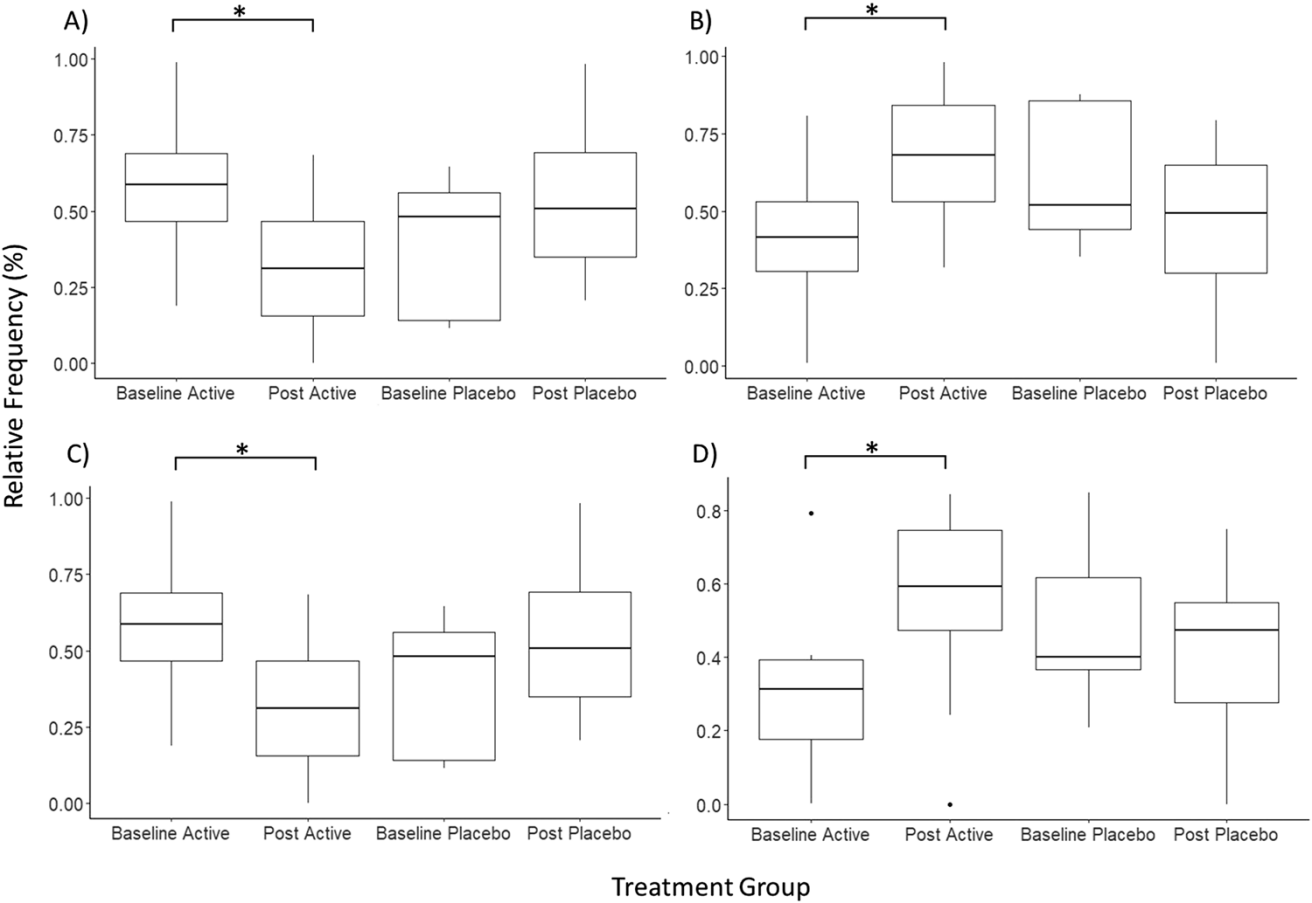
Relative frequency at each treatment stage. The y-axis corresponds to relative frequency of the investigated phylogenetic level (as percentage), and treatment group is presented on the x-axis. Relative frequency is calculated from the assigned sequence variants, filtered to contain only Actinobacteria, and collapsed at each phylogenetic level. The asterisk denotes a significant change (adj*p* ≤ 0.05) between pre and post-RCT groups. (**A**) Bifidobacteriales (**B**) Coriobacteriales (**C**) Bifidobacterium (genus) (**D**) *Collinsella aerofaciens*.

We further investigated Actinobacteria frequency by filtering the dataset to only contain Actinobacteria, and performed statistical analysis at each subsequent phylogenetic level. The relative frequency of Bifidobacteriales decreased by approximately 25% post micronutrient treatment (micronutrient adj*p* = 0.056, placebo adj*p* > 0.20) (Fig. 6A), and this decrease was compensated by an increase of Coriobacteriales (micronutrient adj*p* = 0.057, placebo adj*p* = 0.24), which was not previously observed using ANCOM (Fig. 6B). At the genus level, the decrease in Bifidobacteriales was attributed to *Bifidobacterium* (micronutrient adj*p* = *0*.056, placebo adj*p* = 0.24) (Fig. 6C) where *B*. *longum* and *B*. *adolescentis* were the two major species contributing towards this effect (data not shown). Although no significant change was observed at the family level for Coriobacteriaceae, at the species level the frequency of *Collinsella aerofaciens* significantly increased in the micronutrient group by approximately 20% (adj*p* = 0.01) with no mean change in the placebo group (adj*p* > 0.7) (Fig. 6D).

### Prediction of functional profiles

Analysis performed using PICRUSt identified 328 KEGG Orthology pathways, and analysis performed using Tax4Fun identified 6196 KEGG Orthology pathways. We did not detect significant associations between change in pathway abundance and treatment, after adjustment for multiple testing in either data set (adj*p* > 0.3 and 0.9) (data not shown).

### Correlation between ADHD behaviours and relative abundance of Actinobacteria

**Figure 7:**
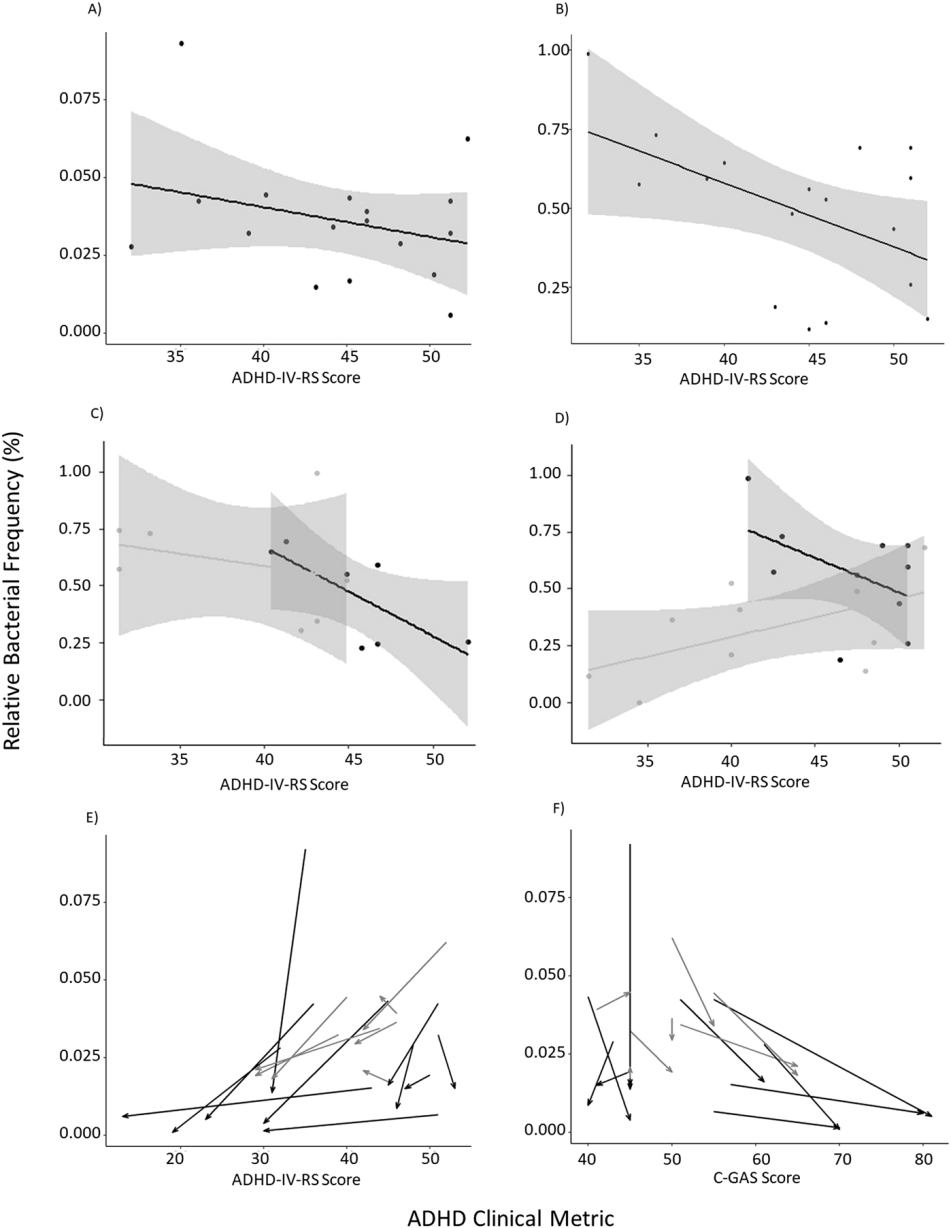
Relative Bacterial Frequency and ADHD measures. The y-axis corresponds to relative bacterial frequency (%) and the ADHD measure is represented on the x-axis. (**A**) Actinobacteria (phylum) plotted against ADHD-IV-RS score for the baseline measurements. (**B**) *Bifidobacterium* (genus) plotted against ADHD-IV-RS score for the baseline measurements. (**C**) *Bifidobacterium* (genus) plotted against ADHD-IV-RS score for the placebo group; black represents the baseline samples and grey represents the post-RCT samples. (**D**) *Bifidobacterium* (genus) plotted against ADHD-IV-RS score for the micronutrient group; black represents the baseline samples and grey represents the post-RCT samples. (**E**) Actinobacteria (phylum) plotted ADHD-IV-RS score for the full dataset, post-RCT and pre-RCT data points from the same participant are connected by arrows, black represents the micronutrient group and grey represents the placebo group. (**F**) Actinobacteria (phylum) plotted C-GAS score for the full dataset,post-RCT and pre-RCT data points from the same participant are connected by arrows; black represents the micronutrient group and grey represents the placebo group.

We next performed a preliminary investigation into the correlation between bacterial abundances and ADHD clinical ratings (C-GAS for global functioning and ADHD-IV-RS for ADHD specific behaviours). This analysis was first performed on the baseline samples (pre-RCT) using relative frequencies from the Actinobacteria filtered frequency table. At the phylum level there was a general trend that suggested increased Actinobacteria may be associated with ADHD-IV-RS; however, this effect did not reach significance (Fig. 7A). When collapsed at the genus level we observed a significant correlation between a higher relative abundance of *Bifidobacterium* and a lower ADHD-IV-RS score (t = −2.3, df = 15, *p* = 0.04) (Fig. 7B), although it should be noted that three outliers are likely to have disproportionately contributed towards this effect. There was no significant correlation with C-GAS, or with the genus *Collinsella* (data not shown). We then further investigated this effect by separately visualising the pre and post-RCT data for the placebo (Fig. 7C) and the micronutrient group (Fig. 7D). Although no significant correlations were observed, the general trend suggested that post micronutrient treatment, a low abundance of *Bifidobacterium* was associated with a low ADHD-IV-RS score (Fig. 7D), which is contradictory to the general trend observed in the pre-RCT and placebo groups.

In an attempt to incorporate the longitudinal data we performed a pairwise repeated measures correlation, which looks at the change between pre and post-time points for each individual. This demonstrated that there was a significant pairwise correlation between a decreased ADHD-IV-RS score and decreased Actinobacteria abundance between pre and post-RCT (*p* = 0.02, r = 0.55) (Fig. 7D), and when assessed against the C-GAS variable (where the higher score is indicative of better functioning) there was also a significant pairwise correlation (r = −0.49, *p* = 0.04) (Fig. 7D). Although a consistent general trend was observed at lower taxonomic levels, including *Bifidobacterium*, this did not meet significance due to low statistical power (data not shown). Furthermore, the potential influence of the micronutrient treatment on this correlation is unclear as we did not have sufficient sample size to incorporate the treatment variable into this analysis. Power analysis was performed using the effect from our weakest correlation (C-GAS, r = −0.43), and suggested a sample size of ~200 would provide ~ 80% power to detect a significant effect with an adjusted *p-*value of 0.05 (adjusted for 5000 tests). However, it should be noted that performing an adjusted statistical model that accounts for treatment, age and BMI will further reduce statistical power.

## Discussion

Our analysis investigated changes in the faecal microbiome composition during a 10-week double-blind RCT comparing micronutrient and placebo treatments. Our pilot study consisted of a subset of children diagnosed with ADHD, who had participated in a 10-week RCT comparing a broad spectrum micronutrient treatment with a placebo^36^. Investigating dietary effects on the microbiome is a growing area of research^10^, and our exploratory research is one of the first microbiome analyses associated with controlled nutrient manipulation in humans, performed using an RCT^11^, and is the first to investigate such changes with respect to ADHD. For each individual, the microbiome was assessed using 16S rRNA gene sequencing on the Illumina MiSeq platform. These preliminary results suggest that micronutrient supplementation may be associated with small, subtle changes in human gut microbiome composition.

We did not detect any substantial change in alpha or beta diversity between the pre and post time-point measures in the micronutrient or placebo group. However, observed OTUs significantly increased in the treatment group, and the magnitude of the change was significantly larger than the placebo group, which showed no mean change. OTUs are a qualitative measure of community richness, and this result suggests that micronutrient treatment may support a more diverse microbiome, or aid in protecting against random fluctuations. A similar change was observed with the Shannon diversity index, which is a quantitative measure of species diversity; however, the effect was not as pronounced. Metrics that incorporated phylogenetic relationships, such as the Faith’s Diversity measures did not significantly differ. Analysis of the raw data indicated that the changes in alpha diversity were largely driven by cumulative changes in low abundance species and this change was therefore not observed at the phyla level (Table 1), and was not detected using relative frequencies. This effect was not likely to have resulted from incorrect attribution of sequence variants, as we excluded features that were observed less than 100 times across samples, which is a higher threshold than is used in most microbiome studies.

We anticipated that the observed modifications in the human microbiome composition were likely to reflect responses to changing metabolic requirements in the micronutrient group. We therefore predicted functional KEGG Orthology pathways using two common bioinformatic algorithms (PICRUSt and Tax4Fun) that rely on metagenomics computation, predicted from the 16S rRNA gene sequences. This approach did not detect a significant association between the micronutrient treatment and the abundance change in predicted functional pathways. This was at least partially due to the small sample size, which substantially reduced statistical power for detecting such changes. Furthermore, 16S rRNA gene sequencing is not an optimal approach for predicting functional changes, as it is based on the presence or absence of a single bacterial gene, from which metagenomics predictions are inferred^53–55^.

We next investigated differential abundances at each taxonomic level using the ANCOM package and also repeated the analysis using pairwise differences. The relative abundance of Actinobacteria significantly decreased in response to micronutrient administration, and this effect was significant at the taxonomic levels of class and order, where changes in Bifidobacteriales appeared to be driving the effect. These observations reinforce the findings from a similar study by Jaeggi *et al*.^11^. Bacteria from the genus *Bifidobacterium* demonstrated the largest decrease in relative abundance, and although 16S rRNA analysis is not well suited to taxonomic classification at the species level, these tentative results suggested that *B*. *longum* and *B*. *adolescentis* were the two major species contributing towards this effect. This decrease in relative frequency was largely compensated by an approximately 20% increase of the genus *Collinsella*, from the order Coriobacteriaceae, which were the only species that significantly increased in the micronutrient group. It should also be noted that at the species level, statistical power was reduced and the absence of these species from some individuals increased variability. Furthermore, pairwise difference analyses were performed on a dataset that was filtered only to contain Actinobacteria, and although this is a common technique, the adjustment for false discovery is less than when the analysis was performed on the full dataset (as with ANCOM).

There is growing evidence to support a role of diet in the regulation of ADHD behaviour, and although it is likely that the gut microbiome plays a significant role^56–58^, to date, there has been very limited scientific investigation into the biological connection. The primary objective of this pilot study was to investigate the potential influence of micronutrient administration on the gut microbiome. This sub-cohort consisted of paediatric patients with ADHD, and in order to predict an appropriate sample size for future studies we performed a preliminary investigation into the correlation between bacterial abundance and the reported ADHD metrics. Due to the small sample size we were unable to incorporate the micronutrient treatment variable into this analysis, although the dataset was filtered to contain only bacteria from the phyla Actinobacteria, as these demonstrated a significant change in response to the micronutrient treatment.

In our dataset we identified *Bifidobacterium* as being of potential relevance to ADHD; however, the outcomes of our results are unclear and appeared contradictory. Overall there appeared to be a general trend supporting the observation that higher *Bifidobacterium* correlated with a lower ADHD-IV-RS score, except for post-micronutrient treatment where a low *Bifidobacterium* abundance was associated with a low ADHD-IV-RS score. Although the treatment likely contributed towards this effect, we did not have sufficient power to incorporate this variable into the analysis, and potential outcomes are unknown. Pairwise repeated measures correlation, which incorporates pre and post-RCT data, did not have the power to detect this effect, although a pairwise decrease in ADHD-IV-RS score was associated with a pairwise decrease in the relative abundance of Actinobacteria at the phyla level. These pilot data suggest a much larger study is warranted, and power analysis indicates a samples size of 200 individuals would be suitable. In the absence of such a large study, these current results should be interpreted with caution.

It is interesting to note that several recent investigations in this area have also identified the genus *Bifidobacterium* as having potential relevance to ADHD;^28–30^ and likewise the outcomes have also been contradictory. Several studies have found *Bifidobacterium* including *B*. *longum* appeared to have a protective effect against developing neuropsychiatric disorders, including ADHD^29,30^, whereas Aarts *et al*.^28^ report a higher abundance of *Bifidobacterium* in ADHD cases compared to controls. Potential causes for these contradictory results are discussed by Aarts *et al*.^28^, and heterogeneity in the diagnosis of neuropsychiatric disorders is likely to be an additional confounding factor in many studies. Furthermore, our analysis was a pilot-study and is therefore prone to bias from the small sample size where large effects in a small number of individuals in each group disproportionately contributed towards the change. Our data also demonstrate the dynamic nature of both ADHD metrics and the microbiome, where regression to the mean could contribute towards contradictory results, and therefore, future studies should incorporate multiple longitudinal time points. Furthermore, the role that the gut microbiome plays in the development of such disorders is unlikely to be directly causative or the result of a single bacterial genus, but instead part of a complex combination of many genera, with both genetic and environmental factors. Although the diet intake questionnaire indicated that there was no significant difference in the recorded dietary habits between pre and post RCT for either group, we cannot eliminate the possibility that the observed changes were related to macronutrient intake^23^ (rather than micronutrient intake), as this is technically difficult to quantify and record.

These findings suggest that micronutrient treatment does not correspond with large scale changes in community composition and structure of the human faecal microbiome content during a 10 week RCT; however, small non-specific changes involving many phyla are likely to have contributed towards a minor community level effect. Although there were no substantial changes at the community level, we did observe a very specific decrease in the abundance of bacteria from the phyla Actinobacteria, which was largely attributed to species from the genus *Bifidobacterium*. Further research is required to establish the role that *Bifidobacterium* contribute towards neuropsychiatric disorders; however, these findings suggest that micronutrient administration could be used as a safe, therapeutic method to modulate *Bifidobacterium* abundance, where required.

## Conclusion

We investigated changes in the composition and structure of the human faecal microbiome content during a 10-week RCT for micronutrient treatment, in a sample of children with ADHD. We did not observe substantial changes in community structure. However, we did detect a significant decrease in the abundance of species from the genus *Bifidobacterium*, which supports previous findings^11^. Currently there are contradictory conclusions in the literature regarding the role that the microbiome plays in neuropsychiatric disorders, and it is interesting that many researchers are identifying *Bifidobacterium* species as key drivers. This preliminary research is suggestive of a mechanistic interaction between Actinobacteria abundance and ADHD, which could have potential implications for modulating and regulating ADHD behaviour. Our pilot study provides an initial observation into this area of research, and highlights an interesting avenue for further investigation in a larger cohort. Furthermore, these novel results validate the need for future research on the biological connection between ADHD, diet and the microbiome^11^.

## Supplementary information

Supplementary File 1

Supplementary File 2

Supplementary File 3

Supplementary File 4.

Supplementary File 5

Supplementary File 1

## Data Availability

All results are available within the manuscript or in the supplementary file, and original scripts used for data analysis are available on request.
