# Author Correction: Human gut microbiome changes during a 10 week Randomised Control Trial for micronutrient supplementation in children with attention deficit hyperactivity disorder

**DOI:** 10.1038/s41598-020-58141-0

**Published:** 2020-01-21

**Authors:** Aaron J. Stevens, Rachel V. Purcell, Kathryn A. Darling, Matthew J. F. Eggleston, Martin A. Kennedy, Julia J. Rucklidge

**Affiliations:** 1 Department of Pathology and Biomedical Science, University of Otago Christchurch, P.O. Box 4345, Christchurch, New Zealand; 2 Department of Surgery, University of Otago Christchurch, P.O. Box 4345, Christchurch, New Zealand; 3 Department of Psychology, University of Canterbury, Christchurch, New Zealand; 4 Mental Health Division, Canterbury District Health Board, Private Bag 4733, Christchurch, New Zealand

Correction to: *Scientific Reports* 10.1038/s41598-019-46146-3, published online 12 July 2019

This Article contains an error in the legend of Figure 2 and Figure 3.

The legend of Figure 2:

**“**Pairwise difference plots for Alpha diversity metrics. Treatment group is represented on the x-axis and pairwise change between pre and post measurements are demonstrated on the y-axis. The asterisk denotes significance (p = 0.05). (A) Pairwise difference comparison in observed OTUs (Alpha diversity). (B) Pairwise difference comparison of community richness using Shannon indices (Beta diversity).”

should read:

“**Figure 2. Pairwise difference plots for Alpha diversity metrics**. Treatment group is represented on the x-axis and pairwise change between pre and post measurements are demonstrated on the y-axis. The asterisk denotes significance (p = 0.05). (A) Pairwise difference comparison in observed OTUs between micronutrient and placebo group. (B) Observed OTUs for each time point of the micronutrient and placebo groups.”

Additionally, the legend of Figure 3:

“Pairwise difference plots for Alpha and Beta diversity metrics. Treatment group is represented on the x-axis and pairwise change between pre and post measurements are demonstrated on the y-axis. The asterisk denotes significance (adjp = 0.05). (A) Pairwise difference comparison in observed OTUs (Alpha diversity). (B) Pairwise difference comparison of community richness using Shannon indices (Beta diversity).”

should read:

“**Figure 3. Pairwise difference plots for Beta diversity metrics**. Treatment group is represented on the x-axis and pairwise change between pre and post measurements are demonstrated on the y-axis. The asterisk denotes significance (adjp = 0.05). (A) Pairwise difference comparison in Shannon indices between micronutrient and placebo group. (B) Comparison of community richness using Shannon indices for each time point of the micronutrient and placebo groups.”

