# Supplementary material for: Human gut microbiome changes during a 10 week Randomised Control Trial for micronutrient supplementation in children with attention deficit hyperactivity disorder
Source: Sci Rep. 2019 Jul 12;9:10128. doi: 10.1038/s41598-019-46146-3 (PMC6625977; doi:10.1038/s41598-019-46146-3)
Supplement: Supplementary file 4 [file 41598_2019_46146_MOESM4_ESM.zip › eaad37e7-960f-4552-aee9-e42ff5bad2b2/data/index.html]

q2\_taxa : barplot
