# Supplementary material for: Human gut microbiome changes during a 10 week Randomised Control Trial for micronutrient supplementation in children with attention deficit hyperactivity disorder
Source: Sci Rep. 2019 Jul 12;9:10128. doi: 10.1038/s41598-019-46146-3 (PMC6625977; doi:10.1038/s41598-019-46146-3)
Supplement: Supplementary file 6 — Supplementary File 1 [file 41598_2019_46146_MOESM6_ESM.pdf]

Human gut microbiome changes during a 10 week Randomised Control Trial for micronutrient supplementation in children with attention deficit hyperactivity disorder.

Aaron J. Stevens[1], Rachel Purcell[2], Kathryn A. Darling[3], Matthew J. F. Eggleston[4], M. A. Kennedy[1], Julia J. Rucklidge[3].*

[1] Department of Pathology and biomedical science, University of Otago Christchurch, P.O. Box 4345, Christchurch, New Zealand.

[2] Department of Surgery, University of Otago Christchurch, P.O. Box 4345, Christchurch, New Zealand.

[3] Department of Psychology, University of Canterbury, Christchurch, New Zealand.

[4] Department of Psychological Medicine, University of Otago, Christchurch, P.O. Box 4345, Christchurch, New Zealand.

[*] To whom correspondence should be addressed. Tel: +(64-3) 364-1222; Email: aaron.stevens@otago.ac.nz

Supplementary Table 1: Daily Essential Nutrients (DEN) List with recommended dietary allowances (RDA) for children given in the same unit.

Ingredients:	1 capsule	12 capsules	Male RDA (4-13yrs)	Female RDA (4-13yrs)
Vitamin A (as retinyl palmitate)	384 IU	4,608 IU	1333-2000	1333-2000
Vitamin C (as ascorbic acid)	40 mg	480 mg	25-45	25-45
Vitamin D (as cholecalciferol)	200 IU	2,400 IU	600	600
Vitamin E (as d-alpha tocopheryl succinate)	24 IU	288 IU	10.5-16.5	10.5-16.5
Vitamin K (as phylloquinone)	6 mcg	72 mcg	55-60	55-60
Vitamin K (as menaquinone-7)	2 mcg	24 mcg	55-60	55-60
Thiamin (as thiamin mononitrate)	4 mg	48 mg	0.6-0.9	0.6-0.9
Riboflavin	1.2 mg	14.4 mg	0.6-0.9	0.6-0.9
Niacin (as niacinamide)	6 mg	72 mg	8-12	8-12
Vitamin B6 (as pyridoxine hydrochloride)	4.7 mg	56.4mg	0.6-1	0.6-1
Folate (as L-methylfolate calcium)*	53.3 mcg	639.6 mcg	200-300	200-300
Vitamin B12 (as methylcobalamin)	60 mcg	720 mcg	1.2-1.8	1.2-1.8
Biotin	72 mcg	864 mcg	12-20	12-20
Pantothenic acid (as d-calcium pantothenate)	2 mg	24 mg	3-4	3-4
Calcium (as chelate)	88 mg	1,056 mg	1000-1,300	1000-1,300
Iron (as chelate)	0.9 mg	10.8 mg	8-10	8-10
Phosphorus (as chelate)	56 mg	672 mg	500-1,250	500-1,250
Iodine (as chelate)	14 mcg	163.2 mcg	90-120	90-120
Magnesium (as chelate)	40 mg	480 mg	130-240	130-240
Zinc (as chelate)	3.2 mg	38.4 mg	5-8	5-8
Selenium (as chelate)	13.6 mcg	168 mcg	30-40	30-40
Copper (as chelate)	0.5 mg	5.8 mg	0.4-0.7	0.4-0.7
Manganese (as chelate)	0.6 mg	7.7 mg	1.5-1.9	1.5-1.6
Chromium (as chelate)	42 mcg	504 mcg	15-25	15-21

Molybdenum (as chelate)	10 mcg	120 mcg	22-34	22-34
Potassium (as chelate)	16 mg	192 mg	3800-4500	3800-4500
Choline bitartrate	36 mg	432 mg	250-375	250-375
Alpha-lipoic acid	33.3 mg	399.6 mg		
Mineral wax	12.5 mg	150 mg		
Inositol	12 mg	144 mg		
Acetylcarnitine (as acetyl-L-carnitine hydrochloride)	4 mg	48 mg		
Grape seed extract	3 mg	36 mg		
Ginkgo biloba leaf extract	2.4 mg	28.8 mg		
Methionine (as L-methionine hydrochloride)	2 mg	24 mg		
Cysteine (as N-acetyl-L-cysteine)	2 mg	24 mg		
Germanium sesquioxide (as chelate)	1.4 mg	16.6 mg		
Boron (as chelate)	0.2 mg	1.9 mg		
Vanadium (as chelate)	0.1 mg	1.0 mg		
Lithium orotate (as chelate)	0.07 mg	0.8 mg		
Nickel (as chelate)	0.002 mg	0.024 mg		

Placebo Formula

Placebo Ingredients	12 capsules (mg)
Fiber Acacia Gum	3600.00
Maltodextrin	4751
Cocoa Powder	48
Riboflavin Powder	1.2

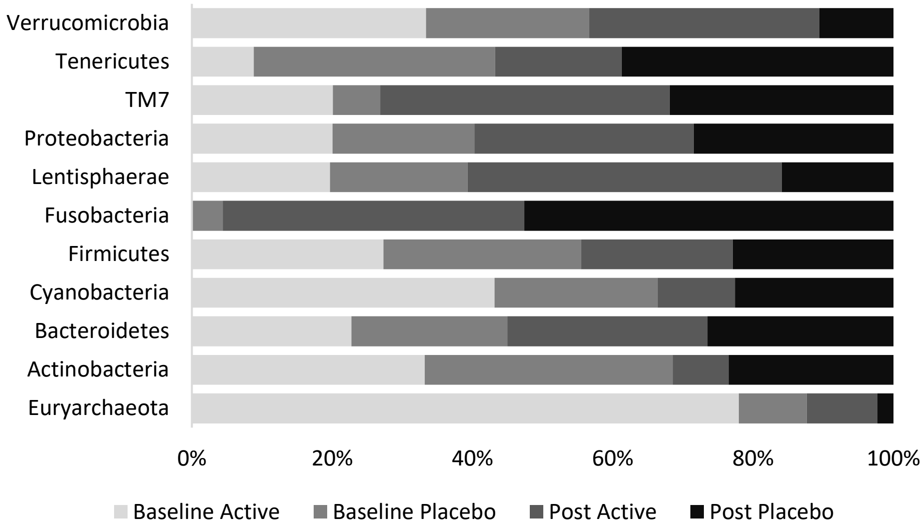

Supplementary Figure 1. The relative frequency of all bacteria detected at the phyla level. Bacterial phylum is represented on the y-axis and relative frequency is represented on the x-axis as percentage per group

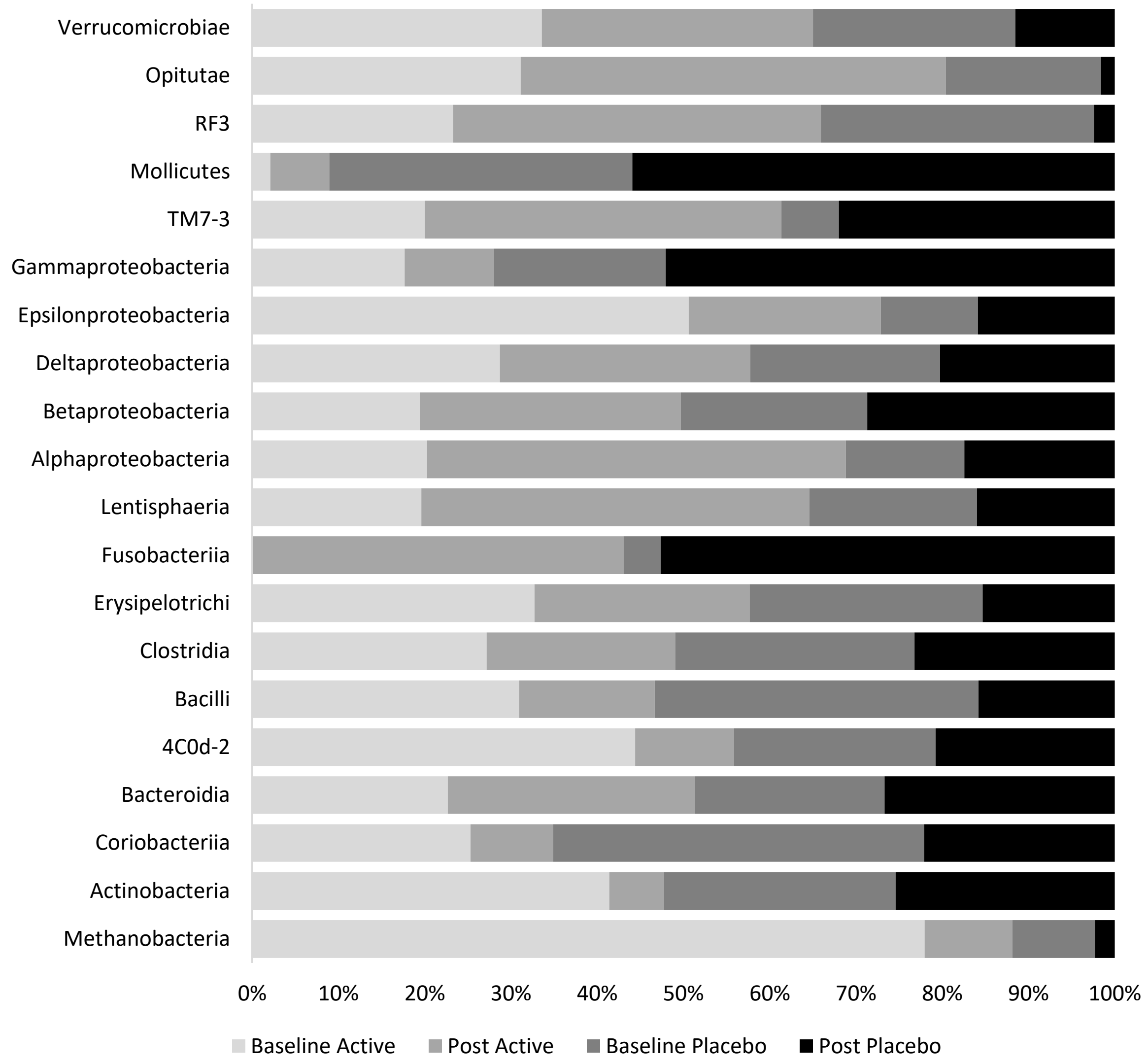

Supplementary Figure 2. The relative frequency of all bacteria detected at the class level. Bacterial class is represented on the y-axis and relative frequency is represented on the x-axis as percentage per group

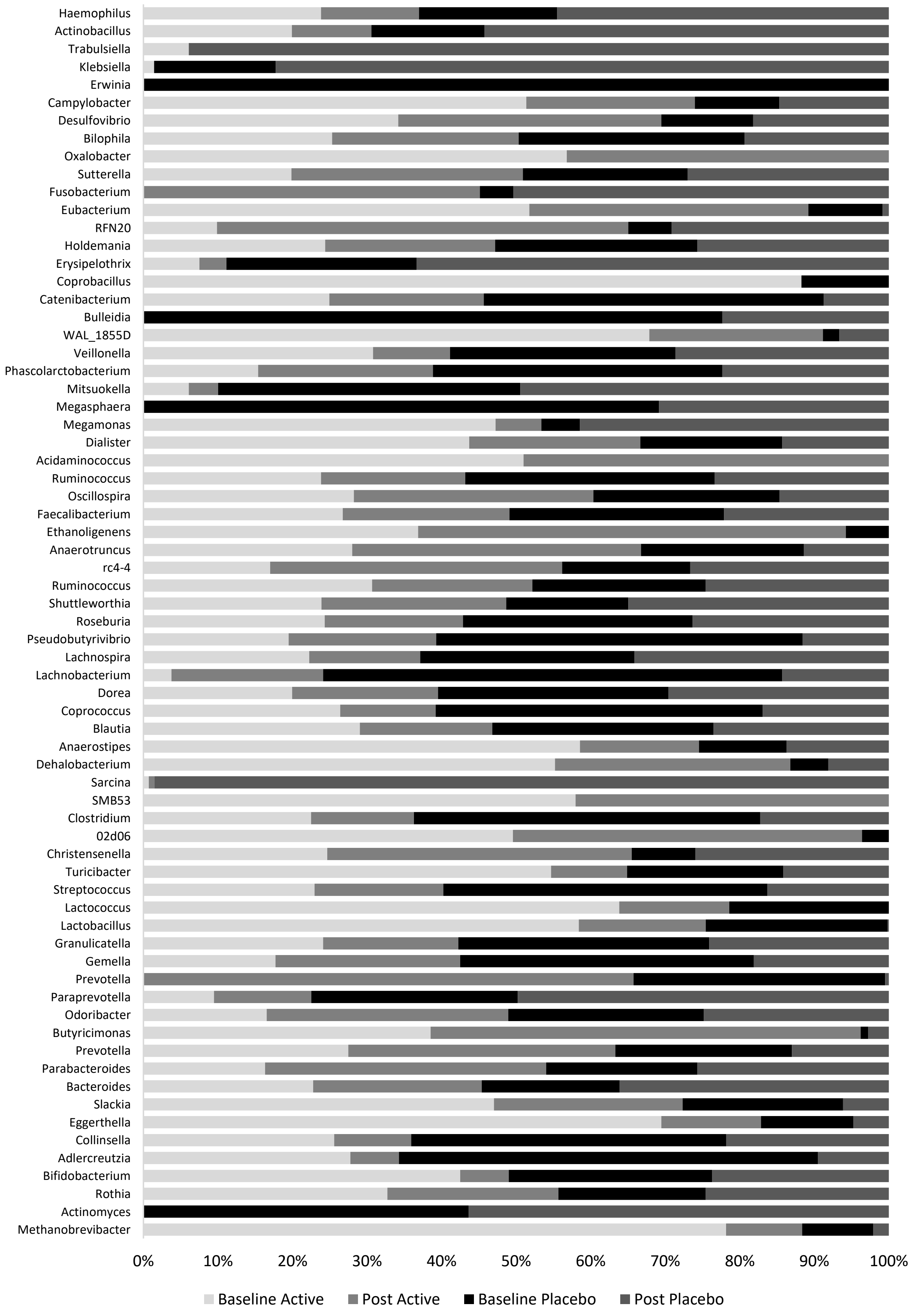

Haemophilus
Actinobacillus
Trabulsiella
Klebsiella
Erwinia
Campylobacter
Desulfovibrio
Bilophila
Oxalobacter
Sutterella
Fusobacterium
Eubacterium
RFN20
Holdemania
Erysipelothrix
Coprobacillus
Catenibacterium
Bulleidia
WAL_1855D
Veillonella
Phascolarctobacterium
Mitsuokella
Megasphaera
Megamonas
Dialister
Acidaminococcus
Ruminococcus
Oscillospira
Faecalibacterium
Ethanoligenens
Anaerotruncus
rc4-4
Ruminococcus
Shuttleworthia
Roseburia
Pseudobutyrivibrio
Lachnospira
Lachnobacterium
Dorea
Coprococcus
Blautia
Anaerostipes
Dehalobacterium
Sarcina
SMB53
Clostridium
02d06
Christensenella
Turicibacter
Streptococcus
Lactococcus
Lactobacillus
Granulicatella
Gemella
Prevotella
Paraprevotella
Odoribacter
Butyricimonas
Prevotella
Parabacteroides
Bacteroides
Slackia
Eggerthella
Collinsella
Adlercreutzia
Bifidobacterium
Rothia
Actinomyces
Methanobrevibacter
0%
10%
20%
30%
40%
50%
60%
70%
80%
90%
100%
Baseline Active
Post Active
Baseline Placebo
Post Placebo

Supplementary Figure 3. The relative frequency of all bacteria detected at the genus level. Bacterial genus is represented on the y-axis and relative frequency is represented on the x-axis as percentage per group.

volatility Results

Metric	shannon
Group column	Treatment
State column	Time
Individual ID column	Subject
Global mean	6.68624
Global standard deviation	0.594139

Control charts

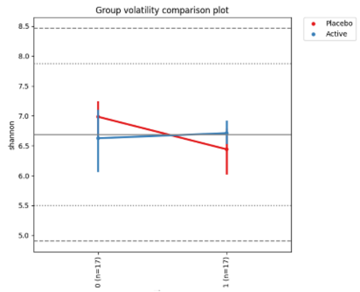

Metric	observed_otus
Group column	Treatment
State column	Time
Individual ID column	Subject
Global mean	401.706
Global standard deviation	63.0563

Control charts

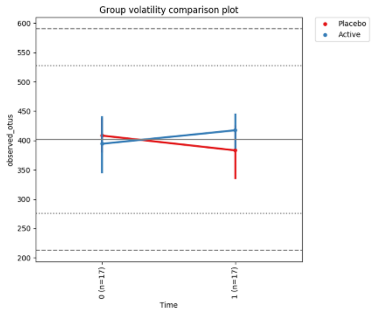

Metric	pielou_e
Group column	Treatment
State column	Time
Individual ID column	Subject
Global mean	0.773824
Global standard deviation	0.0551303

Control charts

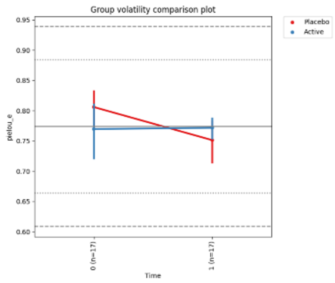

Metric	faith_pd
Group column	Treatment
State column	Time
Individual ID column	Subject
Global mean	24.7045
Global standard deviation	2.86708

Control charts

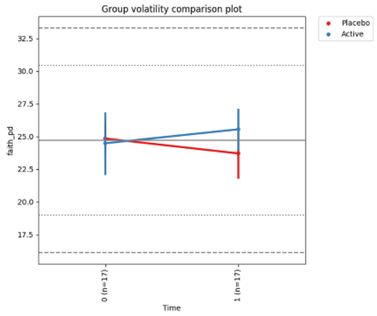

Supplementary Figure 4. Volatility plots generated using qiime2. Change in diversity metrics is represented on the y-axis and sampling time as baseline (Time 0) or end-RCT (Time 1) is represented on the x-axis.
